# The mechanism of delayed release in earthquake-induced avalanches

**DOI:** 10.1098/rspa.2019.0092

**Published:** 2019-07-24

**Authors:** Alexander M. Puzrin, Thierry Faug, Itai Einav

**Affiliations:** 1 Institute for Geotechnical Engineering, ETH Zurich, Stefano-Franscini-Platz 5, 8093 Zurich, Switzerland; 2 Université Grenoble Alpes - Irstea, UR ETGR, 2 rue de la Papeterie BP 76, 38 402 Saint-Martin d'Hères, France; 3 Particles and Grains Laboratory, School of Civil Engineering, The University of Sydney, Sydney, New South Wales 2006, Australia

**Keywords:** earthquakes, snow avalanches, rate-dependent processes

## Abstract

Snow avalanches can be triggered by strong earthquakes. Most existing models assume that snow slab avalanches happen simultaneously during or immediately after their triggering. Therefore, they cannot explain the plausibility of delayed avalanches that are released minutes to hours after a quake. This paper establishes the basic mechanism of delays in earthquake-induced avalanche release using a novel analytical model that yields dynamics consistent with three documented cases, including two from Western Himalaya and one from central Italy. The mechanism arises from the interplay between creep, strain softening and strain-rate sensitivity of snow, which drive the growth of a basal shear fracture. Our model demonstrates that earthquake-triggered delayed avalanches are rare, yet possible, and could lead to significant damage, especially in long milder slopes. The generality of the model formulation opens a new approach for exploring many other problems related to natural slab avalanche release.

## 1. Introduction

That strong earthquakes can trigger snow avalanches is well established [1], with current models predicting immediate release after the seismic event [2–5]. Meanwhile, there have been a few documented examples from Western Himalaya [6] of delayed avalanches that were released minutes to hours after a quake. Non-seismic triggers in these cases were considered unlikely [6], with no snowfall or strong winds occurring in the hours preceding the release, although effects of temperature changes and strong radiation at high altitude cannot be fully excluded. There has also been a debate on whether the deadly avalanche in Italy's Abruzzo region on January 2017 was primarily triggered by snowfalls or by the set of earlier earthquakes [7], the last tremor being 30–50 min before the avalanche. Remarkably, however, in all the reliably documented cases of delayed avalanches, the slopes of the release areas were consistently long, flat and uncommonly mild (from 31 to 33°). Here, we report a general analytical model that establishes the basic physical processes of possible delays in earthquake-induced avalanche release. The main delay mechanism arises from the interplay between strain-rate dependency of snow stiffness [8–10] and strain-rate sensitivity of snow strength [11–15], driving the growth of a basal shear fracture.

**Figure 1.**
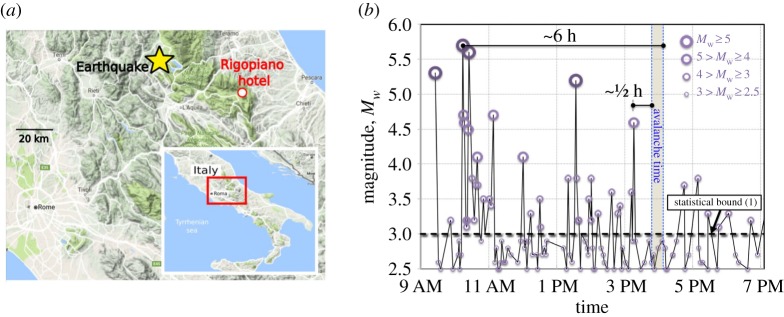
Geographical background of the Rigopiano avalanche. (*a*) Locations of the epicenter of the 18 January 2017 earthquakes in the Abruzzo region, Italy, and of the Rigopiano hotel hit by the avalanche (image from Google Maps). (*b*) The time (GMT) of the avalanche relative to seismic activity in the area, highlighting the longest possible delay of 6 h after the strongest quake and shortest possible delay of ½ h after the closest tremor passing the statistical bound of *M_w_* > 3 described by Podolskiy *et al.* [1]. The grey area in (*a*) highlights the uncertainty of the avalanche time. (Online version in colour.)

The understanding and forecasting of snow avalanches is of major importance in natural-hazard sciences [16]. Unfortunately, the origin of some avalanches cannot be reliably identified or explained by existing snow-avalanche models, which are mostly not designed to capture natural avalanche release. The ‘Rigopiano avalanche’ is one of those cases where the combination of rare conditions inspires further thinking. This avalanche impacted Hotel Rigopiano on 18 January 2017, at the foothill of Monte Siella of Southern Italy's Abruzzo region (figure 1*a*), and literally swallowed the hotel, bulldozing its pieces 10 m down the mountain, killing 29 people and injuring 11.

**Figure 2.**
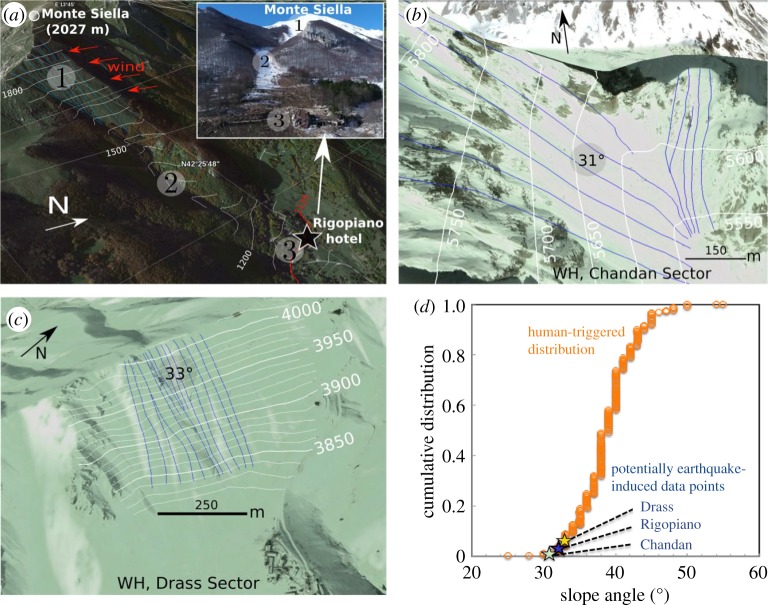
The slopes related to the three potential earthquake-induced avalanches. (*a–c*) The avalanche tracks for the Rigopiano avalanche and Western Himalaya's Drass and Chandan avalanche release zones, respectively (images from Google Earth). Inset in (*a*): image of the avalanche track one month after the Rigopiano avalanche accident, showing the damages caused to the Rigopiano hotel (extracted and adapted from [19]); with (1) release zone; (2) propagation zone; (3) run-out zone. (*d*) Comparison between the slope angles in Rigopiano, Drass and Chandan against the statistics of human-triggered snow avalanches by local skier perturbations (adapted from Schweizer & Jamieson [16]). (Online version in colour.)

Two causal factors were attributed to instigate the avalanche. The first is the strong snowfall and snowdrift in the release zone during the days preceding the avalanche. While we cannot entirely neglect the role of snow accumulation, the likeliness that this has been a causal factor may be questioned in the light of the lack of clear snowstorm-triggered avalanches on the slope of the Rigopiano-avalanche release area. Specifically, at least for over more than 50 years, there has been no record of avalanches of similar magnitude on that slope above the hotel [17], while the region has reportedly experienced other strong snowstorms [18]. Furthermore, this slope (approx. 32°, figure 2*a*; see also the electronic supplementary material) could be regarded as rather mild [2,4,16] with respect to the statistical data of human-triggered avalanches (figure 2*d*).

The second potential factor triggering the Rigopiano avalanche is the series of four major quakes of magnitude *M_w_* > 5 (figure 1*b*) that struck the region before the avalanche, all focused 10 km deep with epicentres just 40 km away (figure 1*a*). The last quake stronger than the statistical bound of *M_w_* = 3 (for epicentres under 150 km away) known to trigger avalanches in the past [1] took place between about 30 and 50 min prior to the avalanche. However, this factor too cannot be reconciled by existing snow-avalanche models, as none of them is able to explain possible delays of avalanches after global seismic perturbations. (The previous attempts to explain delays after local non-seismic perturbations, have led to incoherent paradoxical results [20,21], as discussed in §5 below).

While the chance for the Rigopiano avalanche release can be considered very high even without the earthquakes, this coincidence motivates another look at the unresolved problem of the plausibility of delayed avalanche release. A lack of attention to the possibility of delays may be attributed to the perceived scarcity of such events, which may be partially due to the absence of precise avalanche time records. Nevertheless, over a period of 5 years starting from March 1995, several seismically induced delayed avalanches were identified in the Western Himalaya [6], occasionally devastating lives. The documented delays range from seven minutes to six hours. Strikingly, as highlighted in table 1, the only two documented avalanches, which passed the statistical bound of *M_w_* = 3 for nearby earthquakes, were released from equally long and mild slopes (figure 2*b–d*). Remarkably, these events were not accompanied by any snowstorm on the day before the avalanche. Therefore, these cases reinforce the plausibility that earthquakes may trigger avalanches with a delay.

**Table 1.** Examples for potential earthquake-induced delayed avalanches. Other cases in Western Himalaya (WH) can be found in Singh & Ganju [6], but only those with confirmed delays are listed (slope angles and lengths cannot be found in Singh & Ganju [6], and were established using Google Earth of exact location provided by the authors). Highlighted cases in grey pass the statistical bound by Podolskiy [1].

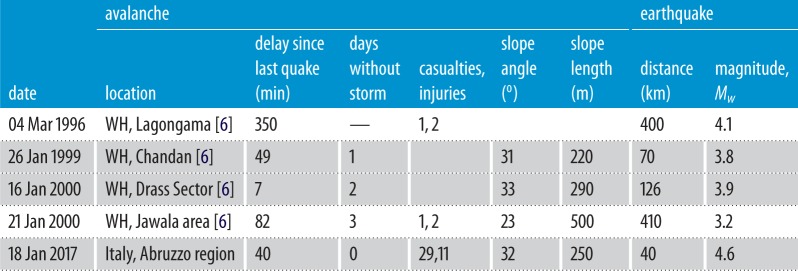

For these reasons, we develop the first model able to: (a) predict the delay of avalanches after earthquakes; (b) explain why avalanches triggered by global seismic perturbations can develop on milder slopes than those by local non-seismic perturbations; and (c) demonstrate why these events are rather rare.

## 2. The mechanism

### (a) Slab release mechanisms

Current snow avalanche models, typically ‘slab-release’ models, assume the presence of a mechanically weak layer as a pre-requisite for an avalanche [2–5,22]. The onset of avalanches is then taken to depend on the strength of the weak snow layer and the growth of cracks in it. Where failure and crack growth in snow depend on competing internal relaxation processes (e.g. slow sintering, bond damage and particle rearrangement), their effects should be considered in slab-release models [23]. Already in the 1930s, Haefeli noted analogies between soil and snow mechanics [24], and the fundamental work of Palmer & Rice [25] has later inspired the development of snow-avalanche [2–5] and soil-landslide models [26,27] alike. In the analysis of landslide mechanisms, slow relaxation processes have explained delayed failures in slowly creeping sub-aerial landslides constrained by obstacles [28,29] and underwater granular landslides [30]. It is, therefore, appealing to explore the delay of avalanches after external perturbations as a function of rate-dependent properties of snow, including strain-rate dependency of stiffness [8–10] and strain-rate sensitivity of strength [11–15]. Strain-rate dependency of stiffness has been considered in analysing the response of a downstream stable snowpack to dynamic loading by a gliding rigid avalanche slab [31] in old snow covers prone to full-depth tensile cracks. On the other hand, whereas strain-rate sensitivity of peak shear strength has been linked to slow ruptures of interfaces [32] and dynamic instabilities during elementary tests in both shear [14,15] and compaction [11,33], this has not been reflected in current slab-release models.

### (b) Strain-rate dependency of shear strength of snow

The key property of snow allowing for the delay in the avalanche release is the rate-sensitivity of the shear strength of the weak snow layer, which first increases with increasing strain rate and then decreases upon a certain critical strain rate. Because of the very fragile nature of snow material, which renders it difficult to handle, mechanical tests on snow samples are extremely challenging to conduct. Tests in compression/extension show that snow exhibits strain-rate hardening followed by strain-rate softening when increasing the strain rate (see, for instance, the pioneering works of Kinosita [12] and Narita [13]). As compression tests are not deviator shear stress-free, they generally support the transitional strain-rate hardening-to-softening scenario for snow under shear. Furthermore, while studies on shear experiments remain scarce (see the summary below) the available datasets clearly confirm that the strain-rate-dependent behaviour observed in compression is also true for the shear strength of snow.

**Figure 3.**
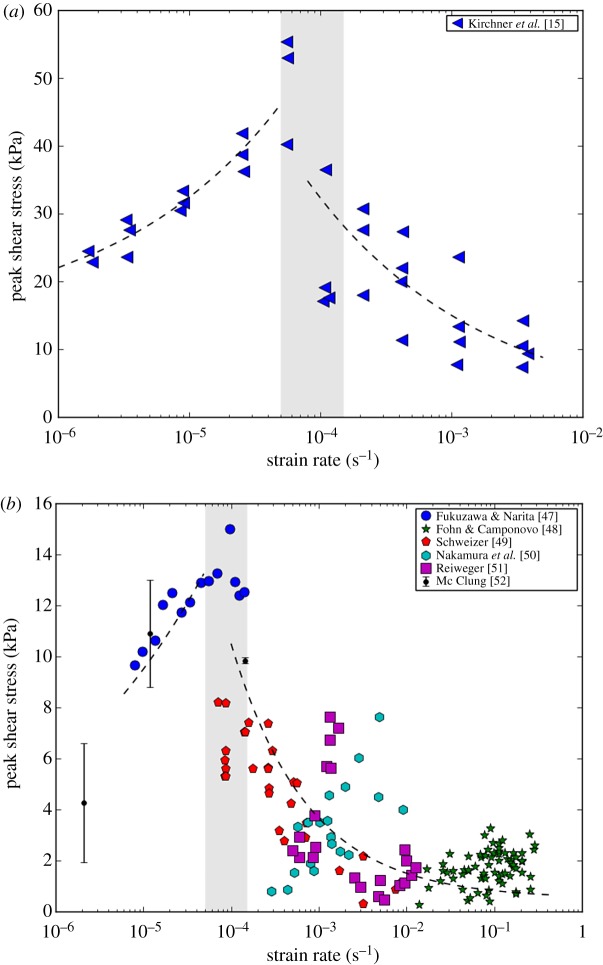
Strain-rate dependence of the peak shear stress of snow. (*a*) A dataset obtained from a single experiment over a broad range of shear strain rates (after Kirchner *et al.* [15]). (*b*) Collation of other datasets from the available literature with tests performed over different smaller ranges of strain rate. The dashed lines indicate the main trends. Note that for the earliest dataset provided by McClung [34] the mean values with standard deviations are shown. (Online version in colour.)

Figure 3 shows the evolution of the shear strength as a function of shear rate, relying on a rather unique dataset [15] obtained over a broad range of strain rate in shear experiments (panel (a)), as well as on the collation of datasets from other shear tests available in literature (panel (b)). The data shown in figure 3*b* include tests on weak layers: depth hoar layer [14], surfaces hoar, faceted crystals, depth hoar or a mixture of them [35] and buried surface hoar [36]. The data also include tests on other types of snow: thin homogeneous snow samples [34], fine-grained snow [15,37] and small rounded particles [38].

The rather unique dataset obtained over a broad range of strain rates (figure 3*a*) clearly shows that strain-rate hardening first occurs at low shear rate (typically smaller than 10^−4^ s^−1^) and is then followed by strain-rate softening at high shear rate (greater than 10^−4^ s^−1^). Compiling all other data available in the literature obtained from shear tests conducted over narrower ranges of strain rate (figure 3*b*) exhibits a rather large scatter, which can be attributed to variations in testing conditions. More studies over a wider range of conditions are required to quantify the transition from hardening to softening for different types of snow with different densities and different temperatures. Nevertheless, the general trend observed in figure 3*a* can also be detected in figure 3*b*. This type of the hardening-softening strain-rate dependency of the shear strength of snow is the key feature of the delayed avalanche release mechanism proposed below.

### (c) The mechanism of delayed avalanche release

**Figure 4.**
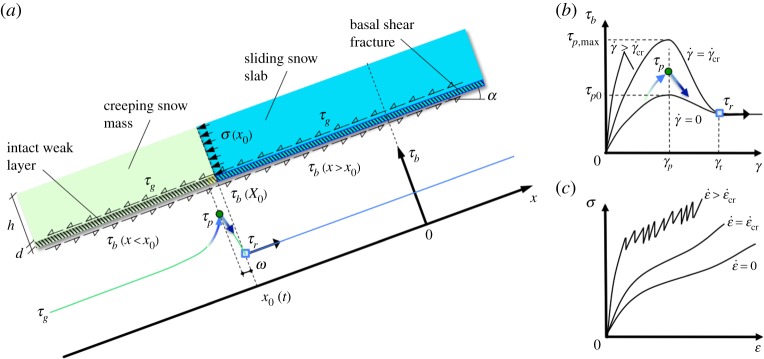
The mechanism underlying the delayed release of earthquake-induced avalanches. (*a*) Geometry of the mechanism and the profile of shear stress $\tau_{b}$ along the weak layer (α: slope angle; ω: length of process zone). (*b*) The snow response during shear, highlighting strain-rate dependency of stiffness (increasing stiffness with strain rate $\dot{\gamma }$, where γ is the shear strain), and strain-rate hardening (increasing peak strength $\tau_{p}$ with $\dot{\gamma }$ up to its maximum value $\tau_{p,\text{max}}$) at low $\dot{\gamma }$, followed by strain-rate softening at higher $\dot{\gamma }.$ (Adapted from Fig. 20 of Schweizer *et al.* [4]). (*c*) The snow response during compression, also highlighting strain-rate dependency of stiffness (σ: normal stress; ε: normal strain; $\dot{\varepsilon }:$ normal-strain rate). (Online version in colour.)

The key mechanism behind our model, as summarized in figure 4, is the interplay between the strain-rate dependency of stiffness of the creeping mass, strain softening of shear strength within the process zone of the basal shear fracture and, most importantly, the strain-rate sensitivity of the shear strength in the intact weak layer. This is novel for both avalanche and landslide analyses and provides the critical ingredient for explaining the delayed-avalanche phenomenon.

The mechanism starts with an earthquake-induced initial ‘basal shear fracture’ of length $l_{0}$ within a pre-existing slope-parallel weak layer (figure 4*a*), where the snow softens to its residual shear strength $\tau_{r}$. Because of this, the unbalanced gravitational sliding force $\tau_{g}-\tau_{r}>0$ in the ‘sliding snow slab’ loads the ‘creeping snow mass’, causing there strain-rate-dependent deformation (figure 4*c*), which in turn drives the process zone of the basal shear fracture into an ‘intact weak layer’. In order to maintain equilibrium, the softening of the shear strength in the process zone is compensated by an increase in peak shear strength in the intact weak layer through rate-hardening (figure 4*b*). The slow growth of the basal shear fracture continues until it reaches a critical strain rate, ${\dot{\gamma }}_{\text{cr}}$, from which point snow exhibits strain-rate softening (figure 3). This critical strain rate determines the critical length $l_{\text{cr}}$ of the shear fracture, at which the peak shear strength reaches its maximum possible value $\tau_{p}=\tau_{p,\text{max}}$ in the intact weak layer. At this moment, no additional resistance can be mobilized through rate-hardening, resulting in the loss of equilibrium and catastrophic propagation of the shear fracture, leading to the release of the avalanche.

In the absence of clear quantification of bond healing effects on $\tau_{r}$ within continuously sheared weak layers, the rate dependence of $\tau_{r}$ is considered negligible to a first approximation. Second-order extensions that take $\tau_{r}$ as rate dependent could be explored in the future, by applying perpetual shear conditions that could be achieved experimentally using the ring shear device. The detection of grain-scale healing would require, however, non-obstructive *in situ* observations that may not be trivial, especially for natural snow. On the other hand, the effect of healing [39] is already considered implicitly in the proposed model (see the following section) through the relaxation time $t_{r}$, which affects both the peak strength $\tau_{p}$ and creep. The compression behaviour (figure 4*c*) of snow exhibits rate-dependent tendencies similar to the shear behaviour [14,15], with very similar relaxation times [11–13]. Here, we are mainly interested in the pre-failure rate-dependent behaviour of the creeping snow mass, because its yield stress is unlikely to be reached before the intact weak layer mobilizes its maximum strength $\tau_{p,\text{max}}$.

## 3. The model

### (a) Problem formulation

In the following, a simplified rate-dependent slab release model is developed, which builds on the mechanism with geometrical and rheological ingredients illustrated in figure 4. Specifically, we assume a symmetric plane strain model of an infinite slope with angle α, where a slope-parallel basal shear fracture of initial length $l_{0}$ forms due to an earthquake in a buried weak layer of thickness *d* at depth *h* (figure 4*a*). The origin of the x-axis is taken in the middle of the initial shear fracture. We shall consider propagation of this shear fracture upslope and downslope focusing on the downslope half of the problem. Initial conditions (before the appearance of the initial shear fracture in the buried weak layer) at the depth *h* are: in the creeping snow mass, an initial internal lateral pressure ($\sigma_{g}$); and in the shear fracture an initial shear stress ($\tau_{g}=\rho gh\sin\alpha$), where ρ is snow density and the initial displacement ($\delta_{g}$) resulting from the long-term decaying creep in the weak layer under the constant initial shear stress $\tau_{g}$. In the following, we adopt a net value $\Delta \tau =\tau_{b}-\tau_{g}$ for the shear stress $\tau_{b}$ in the shear fracture, and the net values $\Delta \sigma =\sigma -\sigma_{g}$ and $\Delta \delta =\delta -\delta_{g}$ for the internal lateral pressure σ and displacement δ of the creeping snow mass, respectively.

**Figure 5.**
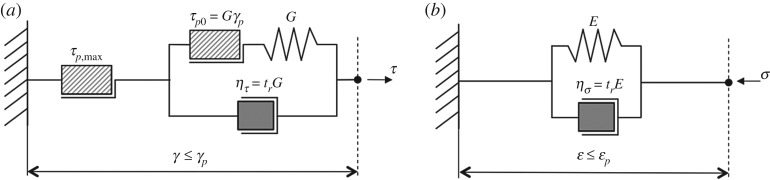
Schematic presentation of pre-failure, rate-dependent constitutive models using rheological elements (the Hookean spring, the Newtonian dashpot and the yield stress slider). (*a*) Kelvin–Voigt shear behaviour with rate-sensitive strength in the intact weak layer; and (*b*) Kelvin–Voigt compressive behaviour in the creeping snow mass.

The fully softened shear fracture (where the strength has been reduced to its residual value, $\tau_{r}$, along its entire length, figure 4*a,b*) is flanked by an intact rate-hardening weak layer where the softening has not yet started. Due to rate-dependent deformations within the rate-hardening weak layer and in the creeping snow mass above it, the shear fracture will propagate parallel to the slope until at a certain time $t_{f}$ after the earthquake it reaches a critical length $l_{\text{cr}}$ and the propagation becomes catastrophic, releasing an avalanche. Here, the rate dependency is accounted only for stiffness and peak strength but not for residual strength and is similar for shear, downslope compression and uphill extension [10]. These pre-failure elements are captured most effectively by employing the simple spring-dashpot-slider models in figure 5*a,b* for the shear ($\tau_{b}$) and normal (σ) stresses, respectively. The stress response of these models is given by the summation of a Newtonian dashpot resistance proportional to the strain rate, and Hookean spring resistance proportional to the strain (until the yield stress is reached in the sliding element). In this classical Kelvin­–Voigt model, the creep decays under constant stress. Unlike the more general Burgers model [10], the Kelvin–Voigt model neglects the possibility of a limited instantaneously pure elastic response (insignificant for our problem as it involves monotonically increasing loading and rather large accumulated deformations) yet avoids non-decaying creep (inconsistent with long-term pre-failure behaviour of snow [10]). Note, that the spring coefficients *G* and *E* in the Kelvin–Voigt models in figure 5*a,b* correspond to the so-called delayed (or long-term) shear and Young's moduli, and not to the instantaneously pure elastic moduli.

While representing a simplification of the real snow behaviour, the models adequately capture the primary phenomena governing the mechanism of delayed avalanche release in the absence of the weak layer collapse [22], i.e. the strain-rate dependency of snow stiffness and the strain-rate sensitivity of its shear strength. Upon shear failure, the snow rapidly loses its strength and the shear stress reaches a rate-independent residual strength value ($\tau_{r}$) (figure 4*b*). It is assumed that this degradation (where the shear strength drops from peak $\tau_{p}$ to residual $\tau_{r}$) occurs within a small process zone whose length $\omega \ll l_{0}$ is at this stage neglected, but can also be explicitly incorporated into analysis similar to the current rate-independent models [40,41].

The length of the shear fracture at time *t* is denoted as $l(t)=2|x_{0}(t)|$, where $l(0)=l_{0}$. Neglecting inertia forces for quasi-static viscosity driven fracture propagation, the equilibrium condition for the creeping mass $x<x_{0}(t)$ is given as:

3.1 $$\frac{\partial \Delta \sigma }{\partial x}=\frac{\Delta \tau }{h};\;\Delta \tau =\tau_{b}-\tau_{g};\;\Delta \sigma =\sigma -\sigma_{g}.$$

The pre-failure $\left(\gamma \leq \gamma_{p}\right)$ shear response of the weak layer can be described schematically using the Kelvin­–Voigt model in figure 5*a*, where for $\gamma <\gamma_{p}$ and $\tau <\tau_{p,\max}$:

3.2 $$\Delta \tau =\frac{G}{d}(\Delta \delta +t_{r}\Delta \dot{\delta });\;\Delta \gamma =\frac{\Delta \delta }{d};\;\Delta \delta =\delta -\delta_{g};\;\Delta \dot{\delta }=\frac{\partial \Delta \delta }{\partial t},$$

where $t_{r}$ is the relaxation time for a quasi-static shearing; *G* is the delayed (long-term) shear modulus and

3.3 $$\delta_{g}=\frac{d}{G}\tau_{g};\;\tau_{g}=\rho gh\;\sin\;\alpha ,$$

where $\delta_{g}$ is the initial displacement of the slab, resulting from the long-term decaying creep in the pre-existing buried weak layer under the constant initial shear stress $\tau_{g}$ (before earthquake).

For $\gamma =\gamma_{p}$ and $\tau <\tau_{p,\text{max}}$ (which is the case for $\Delta \dot{\delta }<\Delta {\dot{\delta }}_{\text{cr}}$), the model reaches the peak strength $\tau_{p}$. The strain-rate dependency of the peak strength is described by a Bingham-type rheology ([12], figures 4*b* and 5*a*):

3.4 $$\tau_{p}(\dot{\delta })=\tau_{p0}\left(1+\frac{t_{r}}{\delta_{p}}\Delta \dot{\delta }\right),\;\text{for }\;\Delta \dot{\delta }<\Delta {\dot{\delta }}_{cr}\;\text{and}\;\delta =\delta_{p}=\gamma_{p}d,$$

where the delayed shear modulus *G* is related to failure parameters via

3.5 $$\frac{G}{d}=\frac{\tau_{p0}}{\delta_{p}}$$

Above a certain velocity $\Delta {\dot{\delta }}_{\text{cr}},$ however, the rate dependency of the peak shear strength has a cut-off given by its maximum value (figures 4*b* and 5*a*):

3.6 $$\tau_{p,\text{max}}=\tau_{p0}\left(1+\frac{t_{r}}{\delta_{p}}\Delta {\dot{\delta }}_{\text{cr}}\right)=\text{const.,}\;\text{for}\;\Delta \dot{\delta }\geq \Delta {\dot{\delta }}_{\text{cr}}\;\;\text{and}\;\delta \leq \delta_{p},$$

from which the critical displacement rate can be back-calculated as:

3.7 $$\Delta {\dot{\delta }}_{\text{cr}}=\frac{\delta_{p}}{t_{r}}\left(\frac{\tau_{p,\text{max}}}{\tau_{p0}}-1\right).$$

The residual shear strength $\tau_{r}$ as well as parameters $\delta_{p}=\gamma_{p}d$ and $\delta_{r}=\gamma_{r}d$ are assumed here to be rate independent.

The rate-dependent compression and extension in the creeping snow mass, which takes place, respectively, down and upslope from the sliding slab (figures 4*c* and 5*b*), is assumed to have the same relaxation time $t_{r}$ as in shear, which is consistent with the findings in [10]:

3.8 $$\Delta \sigma =E^{\prime }\frac{\partial }{\partial x}(\Delta \delta +t_{r}\Delta \dot{\delta });\;\Delta \varepsilon =\frac{\partial \Delta \delta }{\partial x},$$

where $E^{\prime }=E/(1-\nu^{2})$ is the delayed plain strain modulus. Combining equations (3.2) and (3.8) gives:

3.9 $$\Delta \sigma =\frac{E^{\prime }d}{G}\frac{\partial \Delta \tau }{\partial x}.$$

### (b) Stresses in the creeping snow slab

Differentiating equation (3.1) with respect to *x* and substituting equation (3.9) into it gives a differential equation for lateral stresses:

3.10 $$\frac{\partial^{2}\Delta \sigma }{\partial X^{2}}=\Delta \sigma ;\;X=\frac{x}{l_{e}};\;l_{e}=\sqrt{\frac{E^{\prime }hd}{G}},$$

where we introduced the normalized coordinate $X=x/l_{e}$ and the characteristic length $l_{e}$.

Using the boundary conditions (zero stress changes at infinity and equilibrium of lateral stresses at the boundary $x_{0}(t)$ between the creeping snow mass and the sliding snow slab in figure 4*a*):

3.11 $$\Delta \sigma (-\infty ;t)=0;\;\Delta \sigma (x_{0};t)=\frac{\tau_{r}-\tau_{g}}{h}x_{0}(t)=\frac{\Delta \tau_{r}}{h}x_{0}(t),$$

the following solution of equation (3.10) can be obtained:

3.12 $$\Delta \sigma (X;t)=\frac{\Delta \tau_{r}l_{e}}{h}X_{0}(t){\text{e}}^{X-X_{0}(t)},$$

and from equation (3.1) it follows that

3.13 $$\Delta \tau =\frac{\partial \Delta \sigma }{\partial X}\frac{h}{l_{e}}=\Delta \tau_{r}X_{0}(t){\text{e}}^{X-X_{0}(t)}.$$

Equilibrium can be maintained only as long as the maximum shear stress does not exceed the peak maximum strength $\Delta \tau_{r}X_{0}(t)\leq \Delta \tau_{p,\text{max}}$ which provides an expression for the critical length for catastrophic shear fracture propagation:

3.14 $$\frac{l_{\text{cr}}}{2l_{e}}=-\frac{\Delta \tau_{p,\text{max}}}{\Delta \tau_{r}}=\frac{\tau_{p,\text{max}}-\tau_{g}}{\tau_{g}-\tau_{r}},$$

where the peak strength $\tau_{p,\text{max}}$ is given by equation (3.6).

### (c) Displacements in the creeping snow mass

Substituting the shear stress in equation (3.13) into constitutive equation (3.2) gives the equation for the displacements in the creeping snow mass:

3.15 $$\Delta \delta +t_{r}\Delta \dot{\delta }=\frac{\Delta \tau_{r}d}{G}X_{0}(t){\text{e}}^{X-X_{0}(t)}.$$

Note that in spite of the moving boundary invading the creeping mass, the convective derivatives can be neglected because the coordinate system is fixed in space and prior to being invaded by the sliding snow slab the material points in the snow mass only experience small displacements.

The boundary condition for the displacements of the creeping snow mass at the tip of the shear fracture $X_{0}(t)$ is defined by the fact that in order to reach the residual strength $\tau_{r}$ in the softened weak layer, the slip $\delta_{r}=\gamma_{r}d$ has to be achieved at the boundary of the sliding snow slab (figure 4*b*). The boundary condition for the displacements at the boundary of the creeping snow mass is defined by the fact, that in order to reach peak strength $\tau_{p}$ in the rate hardening layer, the slip $\delta_{p}=\gamma_{p}d$ has to be achieved (figure 4*b*). This discontinuity of displacements across the boundary $X_{0}(t)$ is a result of neglecting the length of the process zone of the basal shear fracture, where the stress drops from $\tau_{p}$ to $\tau_{r}$, and does not affect subsequent derivations.

The initial (post-earthquake) condition for displacements in the creeping snow mass follows from neglecting instantaneously pure elastic response in the Kelvin–Voigt model:

3.16 Δδ(x,0)=0.

The solution for equation (3.15) with the initial condition (3.16) depends on the coordinate of the moving boundary $X_{0}(t)$, representing the tip of the shear fracture:

3.17 $$\Delta \delta (X,T)={\text{e}}^{X-T}\frac{\Delta \tau_{r}d}{G}\int_{0}^{T}X_{0}(t){\text{e}}^{t-X_{0}(t)}\text{d}t,$$

where

3.18 $$T=\frac{t}{t_{r}}.$$

### (d) Criterion for the growth of the basal shear fracture

For the basal shear fracture to start growing beyond its initial length $l_{0}=-2X_{0}(0)l_{e},$ the peak strength $\tau_{p}$ has to be reached in the rate hardening intact weak layer at the tip of the shear fracture. This takes place after at the onset time $T_{0}$ when the slip at the tip reaches $\Delta \delta (X_{0}(0),T_{0}\;)=\Delta \delta_{p}=\delta_{p}-\delta_{g}$:

3.19 $$\Delta \delta (X_{0},T_{0})={\text{e}}^{X_{0}-T_{0}}\frac{\Delta \tau_{r}d}{G}\int_{0}^{T_{0}}X_{0}(t)e^{t-X_{0}(t)}\text{d}t=\Delta \delta_{p}.$$

The increase in displacement Δδ in time is caused by the rate-dependent deformation of the creeping snow mass and underlying weak layer under the constant load corresponding to the constant length of the shear fracture $X_{0}(T\leq T_{0})=X_{0}(0)$. Substitution of the $X_{0}(T)=X_{0}(0)$ into equation (3.19) produces

3.20 $$\frac{\Delta \tau_{r}d}{G}X_{0}(0)(1-{\text{e}}^{-T_{0}})=\Delta \delta_{p}.$$

Therefore, since the initial length of the basal shear fracture is $l_{0}=-2X_{0}(0)l_{e}$, the time $t_{0}$ for the onset of its growth can be determined

3.21 $$t_{0}=t_{r}\ln\frac{l_{0}}{l_{0}-l_{g}};\;l_{g}=\frac{2l_{e}}{k};\;k=-\frac{\Delta \tau_{r}d}{G\Delta \delta_{p}}=\frac{(\tau_{g}-\tau_{r})d}{(\delta_{p}-\delta_{g})G}>0,$$

where we introduced the growth-triggering length $l_{g}$ and the stress ratio *k*.

This gives the criterion for the growth of the shear fracture:

3.22 $$\;l_{0}>l_{g}.$$

If this condition is not satisfied, the slip at the tip of the shear fracture will never reach $\Delta \delta_{p}$ and the shear facture will not grow beyond its initial length *l*_0_.

### (e) The growth of the basal shear fracture

Subsequent growth of the shear fracture begins at the onset time $T_{0}$, which after substitution into equations (3.17) and (3.20) provides the initial condition for the subsequent evolution of displacements in the creeping snow mass:

3.23 $$\Delta \delta (X,T_{0})=\Delta \delta_{p}{\text{e}}^{X-X_{0}(0)}.$$

For this initial condition, the solution of equation (3.15) is given by

3.24 $$\Delta \delta (X,T)={\text{e}}^{X(T)-T}\left(\frac{\Delta \tau_{r}d}{G}\int_{T_{0}}^{T}X_{0}(t){\text{e}}^{t-X_{0}(t)}\text{d}t+\Delta \delta_{p}{\text{e}}^{T_{0}-X_{0}(0)}\right).$$

The time evolution of the normalized coordinate $X_{0}(T)$ can be found from the condition, that in the rate hardening intact weak layer at the tip of the shear fracture the slip should remain $\Delta \delta (X_{0},T)=\Delta \delta_{p}$:

3.25 $$\Delta \delta (X_{0},T)={\text{e}}^{X_{0}(T)-T}\left(\frac{\Delta \tau_{r}d}{G}\int_{T_{0}}^{T}X_{0}(t){\text{e}}^{t-X_{0}(t)}dt+\Delta \delta_{p}{\text{e}}^{T_{0}-X_{0}(0)}\right)=\Delta \delta_{p}.$$

or

3.26 $$\frac{\Delta \tau_{r}d}{G\Delta \delta_{p}}\int_{T_{0}}^{T}X_{0}(t)e^{t-X_{0}(t)}\text{d}t={\text{e}}^{T-X_{0}(T)}-{\text{e}}^{T_{0}-X_{0}(0)},$$

which after differentiation with respect to the normalized time *T* gives a differential equation for $X_{0}(T)$, with *k* defined in equation (3.21):

3.27 $$-kX_{0}+\frac{\text{d}X_{0}}{\text{d}T}=1.$$

Using the initial condition (that the initial length $l_{0}$ of the shear fracture is known and stays constant until $T_{0}$):

3.28 $$X_{0}(T_{0})=X_{0}(0)=-\frac{l_{0}}{2l_{e}},$$

which gives the following expression for $X_{0}(T)$:

3.29 $$X_{0}(T)={\text{e}}^{kT}\left(\int_{T_{0}}^{T}{\text{e}}^{-kt}\text{d}t-\frac{l_{0}}{2l_{e}}{\text{e}}^{-kT_{0}}\right)=\left(\frac{1}{k}-\frac{l_{0}}{2l_{e}}\right){\text{e}}^{k(T-T_{0})}-\frac{1}{k}.$$

or in terms of the length of the basal shear fracture:

3.30 $$\begin{array}{rl} l(t) & =l_{0}\;\text{ for }\;t\leq t_{0}=t_{r}\ln\frac{l_{0}}{l_{0}-l_{g}};\;l_{g}=\frac{2l_{e}}{k}\\ \;\text{and}\;l(t) & =(l_{0}-l_{g}){\text{e}}^{k(t-t_{0})/t_{r}}+l_{g}\;\text{ for }\;t>t_{0} \end{array}\}$$

This length should be compared to the critical fracture length $l_{\text{cr}}$ for catastrophic avalanche release, which can be obtained from equation (3.14), or other equilibrium, energy or fracture mechanics based criteria [3–5,21,40,41].

### (f) Conditions for delayed avalanche failure

From equations (3.22) and (3.30), it follows that for the basal shear fracture to grow progressively due to creep, the initial post-earthquake shear fracture length $l_{0}$ has to exceed the growth-triggering length $l_{g}$ but has to be smaller than the critical length $l_{\text{cr}}$ for catastrophic avalanche release:

3.31 $$\;l_{\text{cr}}>l_{0}>l_{g}=\frac{2l_{e}}{k}=2\frac{\delta_{p}G\text{/}d-\tau_{g}}{\tau_{g}-\tau_{r}}\sqrt{\frac{E^{\prime }hd}{G}}.$$

It follows that $l_{\text{cr}}>l_{g}$ is a necessary condition for delayed avalanche failures. Using equations (3.14), (3.31) and (3.5) this condition can be expressed as:

3.32 $$\frac{l_{\text{cr}}}{2l_{e}}=\frac{\tau_{p,\text{max}}-\tau_{g}}{\tau_{g}-\tau_{r}}>\frac{l_{g}}{2l_{e}}=\frac{1}{k}=\frac{\delta_{p}G/d-\tau_{g}}{\tau_{g}-\tau_{r}}=\frac{\tau_{p0}-\tau_{g}}{\tau_{g}-\tau_{r}}.$$

Inequality (3.32) is satisfied if

3.33 $$\tau_{p,\text{max}}>\tau_{p0},$$

which is always the case as is seen from equation (3.6).

It follows that the total time $t_{f}$ of delayed avalanche release after an earthquake can be calculated from equations (3.30) and (3.21) as a sum of two terms, the time $t_{0}$ of the onset shear fracture propagation and the time interval $\Delta t_{\text{cr}}$, during which the shear fracture grows until it reaches the critical length $l_{\text{cr}}$:

3.34 $$t_{f}=t_{0}+\Delta t_{\text{cr}},$$

where

3.35 $$t_{0}=t_{r}\ln\frac{l_{0}}{l_{0}-l_{g}};\;\Delta t_{\text{cr}}=\frac{t_{r}}{k}\text{ln}\frac{l_{\text{cr}}-l_{g}}{l_{0}-l_{g}};\;l_{g}=\frac{2l_{e}}{k}.$$

Note, that for stress ratios k≪1 the first term in equation (3.34) becomes $t_{0}∼k\Delta t_{\text{cr}}$ which is considerably smaller than the second one $\Delta t_{\text{cr}}$. Also note, that if the time of delayed failure $t_{f}$ is known, the length of the initial shear fracture $l_{0}$ can be back-calculated from equations (3.34) and (3.35) providing an opportunity to study the evolution of this initial length under seismic loading.

### (g) Parameters of the model

Following Gaume *et al.* [40], the model parameters can be defined using conventional snow strength and stiffness relationships.

Gravitational shear and normal stresses acting on the weak layer plane:

3.36 $$\tau_{g}=\rho gh\Delta \sin\alpha ;\;\sigma_{n}=\rho gh\Delta \text{cos}\alpha .$$

The peak and residual strength in the weak layer:

3.37 $$\tau_{p0}=c_{0}+\tau_{r};\;\tau_{p,\text{max}}=c_{\text{max}}+\tau_{r};\;\tau_{r}=\sigma_{n}\tan\varphi ;$$

where φ is the angle of internal friction; $c_{0}$ is the rate-independent component of the cohesion; $c_{\text{max}}$ is the maximum cohesion reached at the critical shear strain rate. In the post-peak regime, the cohesion gradually drops to zero, as reflected in the above definition of the residual strength $\tau_{r}$.

Substituting these expressions into equation (3.32), we obtain an expression for the stress ratio:

3.38 $$k=\frac{\text{1}-\tan\varphi /\tan\alpha }{c_{0}/\tau_{g}-(1-\tan\varphi /\tan\alpha )}.$$

It follows that the critical growth-triggering length $l_{g}$, which the initial shear fracture has to exceed to trigger the delayed avalanche release, can be expressed as

3.39 $$\frac{l_{g}}{2l_{e}}=\frac{1}{k}=\frac{c_{0}/\tau_{g}}{\text{1}-\tan\varphi /\tan\alpha }-1;\;l_{e}=\sqrt{\frac{E^{\prime }hd}{G}}.$$

According to equation (3.14), which is equivalent to the one presented in Gaume *et al.* [40], the critical length of the shear fracture for catastrophic avalanche release is given by:

3.40 $$\frac{l_{cr}}{2l_{e}}=\frac{c_{\text{max}}/\tau_{g}}{\text{1}-\tan\varphi /\tan\alpha }-1>\frac{l_{g}}{2l_{e}}.$$

From the typical parameter ranges presented by Gaume *et al.* [40], it can be deduced that $c_{0}/\tau_{g}∼10^{0}$, which upon substitution into equation (3.38) gives a quick assessment for the stress ratio

3.41 k≈tan⁡α/tan⁡φ−1

## 4. Parametric and case studies

### (a) The growth of the shear fracture and the total time of delayed release

Our new model enriches previous snow slab-release models with essential rheology, acknowledging both the rate dependency of snow stiffness and the rate-sensitivity of its strength. We follow the equilibrium approach, which has been adopted in fundamental modelling of interfacial dynamics [32], and take the shear fracture as the driver of frictional motion. In doing so, we arrived at solving three differential equations with closed-form analytic solutions for the normal stress σ and displacement δin the creeping snow mass and, finally, for the growing length *l* of the basal shear fracture. Specifically, the growth of this length with time, *t*, is given as

**Figure 6.**
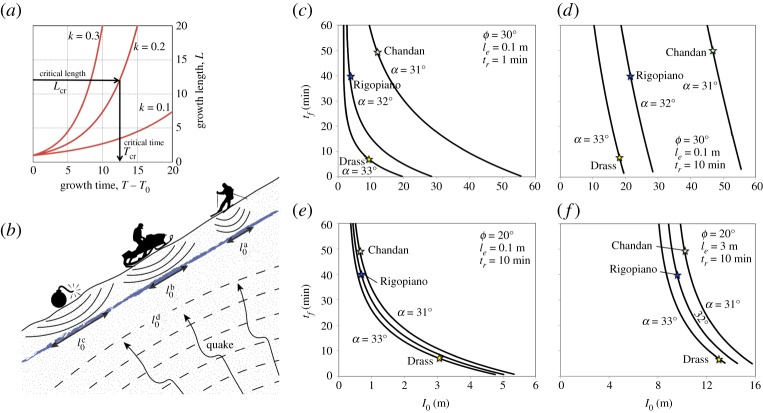
Predicting delays. (*a*) Growth of the rupture plane for different *k* values. (*b*) Dependence of initial fracture length $l_{0}$ on local (explosives, snowmobile and skiers) and global triggers (earthquake). (*c–f*) Sensitivity analysis of initial fracture lengths $l_{0}$ for the three potentially earthquake-triggered delayed avalanches, covering the limits of a wide range of realistic parameters. (Online version in colour.)

4.1 $$\begin{array}{rl} l(t) & =(l_{0}-l_{g}){\text{e}}^{k(t-t_{0})/t_{r}}+l_{g}\\ t_{0} & =t_{r}\ln\frac{l_{0}}{l_{0}-l_{g}}, \end{array}\}$$

which depends on the relaxation time $t_{r}$ of snow, the time for the onset of fracture growth $t_{0}$, and two important physical quantities. The first is the stress ratio *k*,

4.2 $$\begin{array}{rl} k & =\frac{\tau_{g}-\tau_{r}}{\tau_{p0}-\tau_{g}}\\ \tau_{g} & =\rho gh\sin\alpha , \end{array}\}$$

which depends on the residual shear strength $\tau_{r}$, the snow peak strength at zero velocity $\tau_{p0}$ and the snow density ρ (g is the Earth's gravitational acceleration). It follows that for an accelerating growth of the basal shear fracture the gravitational shear stress $\tau_{g}$ has to be larger than the residual strength $\tau_{r}$ but smaller than the peak strength $\tau_{p0}$. Therefore, milder slopes α, for which $\tau_{g}$ is smaller and *k* lower, exhibit slower growth of the shear fracture and are prone to longer avalanche delays (figure 6*a*).

The second quantity is the critical growth-triggering length $l_{g}$:

4.3 $$\begin{array}{rl} l_{g} & =\frac{2l_{e}}{k}\\ l_{e} & =\sqrt{\frac{E^{\prime }hd}{G}}, \end{array}\}$$

defined using the delayed plane strain modulus $E^{\prime }=E_{\text{sl}}/(1-\nu_{\text{sl}}^{2})$, with $E_{\text{sl}}$ and $\nu_{\text{sl}}$ being delayed Young's modulus and Poisson's ratio of the snow slab, respectively; $G=G_{\text{wl}}$ is the delayed shear modulus in the intact weak layer. All these parameters are clearly defined and measurable [10].

Similar to rate-independent models, for immediate avalanche release, our mechanism requires an initial shear fracture length longer than critical, $l_{0}>l_{\text{cr}}$. For delayed avalanches, $l_{0}$ has to be larger than the critical growth-triggering length $l_{g}$ ($l_{g}<l_{0}<l_{\text{cr}}$). On the other hand, if $l_{0}<l_{g}$, our model predicts no fracture growth and only limited deformation, which overcome inconsistencies of previous attempts (see §5).

The relationship between the total time $t_{f}$ of delayed avalanche release and the length of the initial shear fracture $l_{0}$ is given by equations (3.34) and (3.35):

4.4 $$t_{f}=t_{r}\ln\frac{l_{0}}{l_{0}-l_{g}}+\frac{t_{r}}{k}\text{ln}\frac{l_{\text{cr}}-l_{g}}{l_{0}-l_{g}}.$$

The model parameters affecting this relationship can be calculated using equations (3.39)–(3.41):

4.5 $$l_{\text{cr}}=l_{g}+2l_{e}\left(1+\frac{1}{k}\right)s;\;l_{g}=\frac{2l_{e}}{k};\;k\approx \tan\alpha /\tan\varphi -1;\;s=\frac{c_{\text{max}}-c_{0}}{c_{0}}.$$

### (b) Typical ranges of the model parameters

In order to evaluate the model parameters in equations (3.37), three properties of the snow are essentially needed: the friction angle φ, the cohesion *c* and the length $l_{e}$. The latter depends on Young's modulus and Poisson's ratio of the slab, the shear modulus of the weak layer, and the slab and weak layer thicknesses.

It is difficult to determine precise values of the above snow properties for the cases considered in the present study (the Rigopiano case in Italy and the Chandan and Drass cases in India) because they were not measured. Note that this is often the case after avalanche events that took place in non-surveyed mountain remote areas and/or during snowstorm weather conditions. Moreover, snow properties are extremely variable even for a relatively well-documented single event at one given site. In the following, we rather propose a broad range of realistic values for each snowpack property above, guided by the existing literature about the mechanical properties of snow.

#### (i) Friction angle

There have been some attempts to evaluate the internal friction angle of snow weak layers. Detailed studies were recently conducted by Podolskiy *et al.* [42] who reported values of φ ranging from 15° to 30°, and by Reiweger *et al.* [43] who arrived at a mean value of about 20° with values ranging from 12° to 28°. Note that the recent theoretical studies addressing the problem of avalanche release used different values of friction angle of either 20° [41] or 30° [40]. For their recent model for dynamic anticrack propagation in snow, Gaume *et al.* [22] have chosen an intermediate friction coefficient of 0.5 (φ≈ 26.5°). Acknowledging the variability of the friction angle of snow, we considered a friction angleφ ranging from 20° to 30°, in accordance with the literature mentioned above.

#### (ii) Snow cohesion

The snow cohesion is reported to generally range from 0.5 to 2.5 kPa (see [40]) and relevant references therein). Assuming that the maximum cohesion reached at the critical shear strain rate, $c_{\text{max}}$, can be up to five times greater than the rate-independent component of the cohesion, $c_{0}$, this gives a typical range between 0.5 and 4 for the parameter *s*.

#### (iii) Characteristic length $l_{e}$

The characteristic length $l_{e}$ is defined as $l_{e}=\sqrt{E^{\prime }hd\text{/}G}$, where $E^{\prime }=E_{\text{sl}}/(1-\nu_{\text{sl}}^{2})$ is the delayed plane strain modulus of the snow in the slab; $G=G_{\text{wl}}$ is the delayed shear modulus of the snow in the weak layer. Expressing $E^{\prime }$ as the delayed shear modulus of the snow in the slab: $E^{\prime }=2G_{\text{sl}}/(1-\nu_{\text{sl}})$ produces $l_{e}=\sqrt{\left(G_{\text{sl}}\text{/}G_{\text{wl}})(2hd/(1-\nu_{\text{slab}})\right)}$. By adopting a broad range of characteristic lengths $l_{e}$ between 0.1 and 3.0 m, we accommodate typical values of slab thickness *h* (from 0.5 to 5 m); weak layer thickness *d* (from 0.5 to 5 cm); Poisson's ratio of the slab $\nu_{sl}$ from 0.1 to 0.4 (defined in [8,23,40]) and the ratio between the delayed shear moduli $G_{\text{slab}}\text{/}G_{\text{wl}}∼10^{0}-10^{1}$ (assumed to have a similar range as the ratio between elastic moduli [44–46]).

#### (iv) Relaxation time

**Figure 7.**
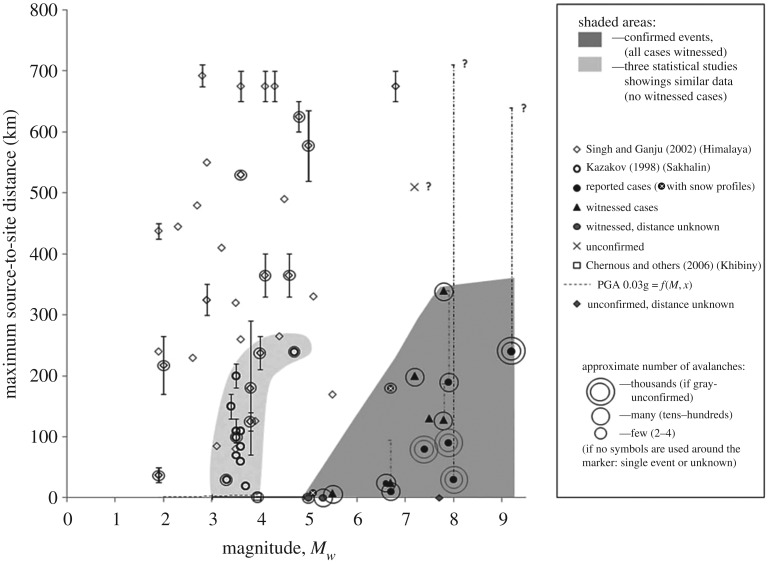
Seismic parameters of earthquake-triggered avalanches. The figure is adopted from Podolskiy *et al.* [1].

Shinojima [10] measured relaxation times in quasi-static torsion, compression and elongation tests. The times were similar in all the test types and varied between 40 s and 40 min, approximately inversely proportional to the strain rate in the range. For the strain rates relevant to our problem (i.e. below and around the critical shear strain rate of about 10^−4^ s^−1^ in figure 7), the relaxation time of 1–10 min appeared to be an adequate choice.

### (c) Parametric study and plausibility of delayed avalanches in India and Italy

The proposed mechanism can be used to demonstrate the plausibility of delayed avalanches, the importance of mild and long slopes for such events, and the scarcity of delayed avalanches. With respect to the plausibility, our model does not impose a theoretical upper bound for the longest possible delay time for arbitrary conditions. Even in steep slopes (where stress ratio *k* is large), there could be indefinitely long delays, if $l_{0}$, caused by the external perturbation, is larger and sufficiently close to $l_{g}$. For mild slopes, with smaller *k*, significant delays are possible even for $l_{0}$ being closer to $l_{\text{cr}}$ than to $l_{g}$.

By considering a broad range of typical snow properties, the corresponding ranges of model parameters estimated above can be summarized as:

4.6 $$l_{e}=0.1-3.0\;\text{m};\;\varphi =20^{\circ }-30^{\circ };\;s=0.5-4.0;\;t_{r}=1-10\;\text{min}$$

For these ranges and mild slopes ($\alpha =31^{\circ }-33^{\circ }$) of observed avalanches in Italy and India, we back-calculate the length of the initial shear fracture $l_{0}$ that could cause the recorded delay times of $t_{f}=5-50\;\text{min}$ (figure 6*c–f*). The resulting initial fracture lengths of $l_{0}=1-100\;\text{m}$ appear to be consistently smaller than the lengths of the potential avalanche initiation zones (reported in table 1), and are thus geographically allowable. This is also consistent with previous results [40,41] that in the absence of the weak layer collapse [22], in mild slopes with inclination close to the friction angle, only long initial basal shear fractures can result in an avalanche. Local triggers by skiers and explosions cannot generate such long fractures. By contrast, a global trigger like an earthquake, with a relatively flat slope-parallel shear wavefront arriving from a large distance, can easily generate long shear fractures within a weak layer in a relatively uniform mild slope (figure 6*b*). This is consistent with the observation that Italian and Indian avalanches took place in mild long slopes between 31° and 33°.

This analysis also explains the rarity of the observed delays in avalanche release. Indeed, such an event requires a combination of rare conditions, such as long mild slopes, thick snow covers and an earthquake of sufficiently high intensity. Nevertheless, it is possible that there are more delayed avalanches than reported due to the absence of precise avalanche time records. Furthermore, in spite of this low probability of occurrence, the hazard of delayed avalanches cannot be ignored due to potentially high damage.

### (d) Effects of the earthquake magnitude and source-to-site distance

The proposed mechanism can also be used to understand effects of the earthquake intensity on the plausibility of delayed avalanches. Podolskiy *et al.* [1], plotted observed seismic events that have potentially caused avalanches (figure 7). The dark grey area in the plot with the magnitude $M_{w}>5$ indicates events confirmed by witnesses, where the avalanche took place during or immediately after the earthquake. The light grey area with the magnitude $3<M_{w}<4.5$ and the epicentral distance with the magnitude R<250 km indicates seismic events whose link to the corresponding avalanches has only been established statistically, without direct observations. The three delayed avalanches investigated in this paper belong to this light grey range (table 1), suggesting that in addition to the lack of direct observation, delayed release could be one of the reasons behind the uncertainty when trying to link between an earthquake and subsequent avalanches. For the avalanches outside of the two grey areas, no link to seismic events could be established.

Our mechanism provides a possible explanation why delayed avalanches occur after earthquakes of moderate magnitude. Indeed, the length $l_{0}$ of the initial fracture increases with the increasing earthquake intensity, and for high magnitudes is likely to exceed the critical length $l_{\text{cr}}$, causing an immediate avalanche release. By contrast, for lower magnitudes, the length $l_{0}$ is likely to be smaller than the growth-triggering length $l_{g}$, necessary for initiating the basal fracture growth. It follows that delayed release is possible only in a certain intermediate range of earthquake intensities, which is qualitatively consistent with the statistical study presented in figure 7.

## 5. Summary and discussion

### (a) Summary of the proposed mechanism

Our model quantifies the mechanical process of delayed release of earthquake-induced avalanches in the following way:

1. Seismic loading creates an initial shear fracture of length $l_{0}$ within an existing weak layer.
2. Appearance of this fracture will redistribute the forces in the sliding slab and will trigger a Kelvin–Voigt type behaviour in the creeping snow mass and in the intact weak layer outside of the shear fracture.
3. This Kelvin–Voigt creep is decaying and if the initial length $l_{0}$ is smaller than the newly defined growth-triggering length $l_{g}$, this decaying creep will not generate sufficient displacement to enable the fracture growth.
4. However, if $l_{0}>l_{g}$, the same decaying creep will produce enough displacement in the intact weak layer to trigger its visco-plastic response. At this moment $t_{0}$, the shear fracture starts growing with the rate of its growth being mainly controlled by the visco-plastic rate-hardening of the weak layer.
5. If the shear strength was rate-independent, the propagation of the shear fracture would become catastrophic already at $t_{0}$. Instead, the rate-hardening allows for a slowly accelerating growth of the shear fracture until a certain critical strain rate is reached in the weak layer.
6. Upon this critical strain rate, the visco-plastic rate-hardening of the shear strength in the weak layer switches to rate-softening. At this moment $t_{f}=t_{0}+\Delta t_{\text{cr}},$ the shear fracture has reached its critical length $l_{\text{cr}},$ which manifests the onset of its catastrophic propagation leading to the avalanche release.

### (b) Significance of adequate constitutive modelling

The few known attempts found in the literature to model viscous shear fracture growth in dry snow did not provide reliable assessment of delays. The first attempt by McClung [20] was made to explain the delay of avalanche release after explosions (by introducing time-dependent elastic parameters into a time-independent fracture energy criterion), though this line of thought has not been taken further. The result exhibits two significant inconsistencies: (1) shorter initial shear fractures produce shorter delay times (with a minimum delay for zero initial shear fracture length); (2) all shear fractures should grow irrespective of their initial length, thus implying orders of magnitude more avalanches than observed with or without explosions. These paradoxes originate from the use of non-decaying Maxwell creep employed for the snow mass and the rate-independent rigid-plastic behaviour taken for the weak layer. In contrast to this model above, our model is based on Kelvin–Voigt decaying creep and captures strain-rate hardening, thus yielding a new expression for the critical length $l_{g},$ below which no shear fracture can start growing. In addition, we find consistently shorter delay times for longer initial shear fractures. The second attempt was made by Bader & Salm [21] and focused on the dynamics of shear fracture propagation in snow but they too employed oversimplified constitutive assumptions in the form of Newtonian fluid with finite viscosity for the snow mass and zero viscosity for the basal shear fracture. In using zero viscosity for the shear fracture, the continuity conditions declared for the normal stresses and normal velocities could not be satisfied. A number of other significant flaws followed from the Newtonian fluid assumptions: (1) as criticized by Schweizer [23], their model predicts thinner weak layers to be more prone to failure than thicker ones (with unlimited failure of all shear fractures for infinitesimal thickness d→0, irrespective of their initial length $l_{0}$); (2) probable shear fracture propagation for slopes milder than the snow's friction angle; and finally (3) indefinitely large displacements, even for very short initial fractures and very mild slopes. Again, our model is clean from such flaws thanks to the use of self-consistent and more realistic constitutive assumptions.

### (c) Generality of the model formulation

The proposed model is not limited to earthquake-induced or delayed avalanches. The generality of its formulation has enabled us to construct a most complete model of natural slab avalanche releases. It is, therefore, proposed that with minimal constitutive refinements, our formulation may dramatically improve our understanding of many other problems related to slab avalanches and triggering processes.

## 6. Conclusion

In conclusion, our model demonstrates that delayed avalanches, while plausible, require a combination of rare conditions, such as long mild slopes, thick snow covers and an earthquake of sufficiently high intensity. Nevertheless, it is possible that there are more delayed avalanches than reported due to the absence of precise avalanche time records. Furthermore, in spite of this low probability of occurrence, the hazard of delayed avalanches cannot be ignored due to potentially high damage.

Our basic physical model paves the path for better forecasting delayed snow avalanches, and highlights the (previously ignored) risk of delays in milder slopes. In addition to the easily accessible slope angles, a full forecast requires three lengths: the growth-triggering length $l_{g}$, the critical length *l*_cr_ and the initial length $l_{0}$ of the basal shear fracture. Where expressions for $l_{g}$ and *l*_cr_ were derived, they depend on a number of well-defined physical parameters varying with temperature and density. By contrast, the way $l_{0}$ depends on the earthquake magnitude and snow conditions is a well-recognized uncertainty [1] and requires further work. However, the framework proposed here provides a relation between the time of delay and the initial fracture length. Accordingly, careful attention should be paid to registering the exact time of future potentially earthquake-induced avalanches, as this would facilitate a better understanding of the evolution of the initial basal shear fractures during seismic loading.

## Supplementary Material

Supplementary Material

## Supplementary Material

Data in Fig1B

## Supplementary Material

Data for Chandan in Fig2

## Supplementary Material

Data for Drass in Fig2

## Supplementary Material

Data for Rigopiano in Fig2

## Supplementary Material

Data in Fig2D

## Supplementary Material

Data in Fig3A

## Supplementary Material

Data in Fig3B

## Supplementary Material

Data in Fig6A

## Supplementary Material

Data in Fig6C-F

## Data accessibility

All data needed to evaluate the conclusions in the paper are present in the paper and the electronic supplementary material.
