# Supplementary material for: The mechanism of delayed release in earthquake-induced avalanches
Source: Proc Math Phys Eng Sci. 2019 Jul 24;475(2227):20190092. doi: 10.1098/rspa.2019.0092 (PMC6694300; doi:10.1098/rspa.2019.0092)
Supplement: Supplementary Material [file rspa20190092supp1.pdf]

SUPPLEMENTARY MATERIAL

a. Slope angles of release zones in Italian and Indian (Western Himalaya) cases

Local slopes along the Rigopiano avalanche release zone in Italy (see zone 1 in Fig. 2A in Main Text) and the Chandan (Fig. 2B) and Drass (Fig. 2C) avalanche release zones in Western Himalaya, India, were first calculated for every 25 m long segments along a number of profiles (of about 600 m in length for Rigopiano, 525 m for Chandan and 400 m for Drass) uniformly distributed over the entire width of the release zone, using the topographical data available from Google Earth. Those data sets were then used to calculate the average slope of each release zone and the corresponding length. We used the following criterion to calculate the corresponding (most probable) length of the release zone: the difference between the average slope angle and the (local) slope angle of any 25 m long segment never exceeds 2° along each single profile considered. The method yields:

- an average slope of 29.5° for the entire surface of the release area in Rigopiano (42°25'39" N 13°45'41" E), and 32° for the upper (steepest) part corresponding to the first 250 m from the top of the release zone. The value of 32° was obtained by considering the profiles in the south part only of the entire potential release area and is fully consistent with the analysis recently proposed by Chiambretti et al. (2018) who concluded that the 18th January 2017 avalanche in Rigopiano may have started in the south part of the release zone. Note that considering all the profiles defined in Fig. 2A (Main Text) over the entire width of the potential release area gives a smaller slope angle equal to 30.5°, given the fact that the north part of the entire potential release area is milder than the south part.
- an average slope of 28.5° for the entire surface of the release area in Chandan (35°07'50"N 77°08'30" E), and 31° for the steepest part (most probable release zone) corresponding to the first 220 m below the rocky zone (see Fig. 2B in Main Text). In want

of any information about the exact location of the release zone, note that another (less probable) potential release zone could have been defined, as depicted by the area B shown in Fig. S1. As this area B has an average slope of 31° (and a corresponding length of about 160 m) which is equal to the average slope calculated for area A, this does not change the conclusions.

- an average slope of 32° for the entire surface of the release area in Drass (34°28’37” N 75°42’36” E), and 33° for the upper (steepest) part corresponding to the first 290 m from the top of the release zone.

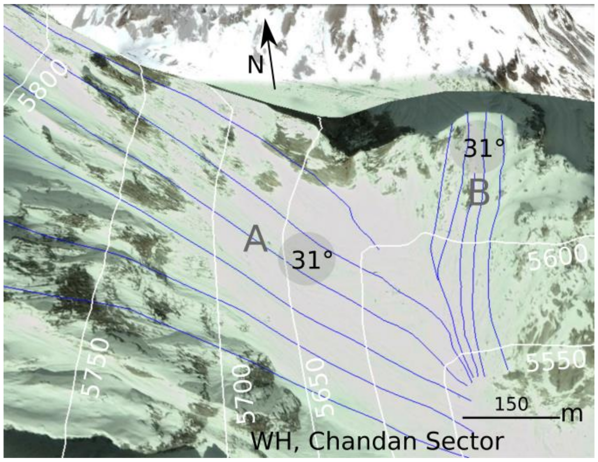

Fig. S1. Avalanche release zone in Chandan sector, Western Himalaya. The area B could be identified as another potential release area, but less probable than area A. Note that the average slope of that area B was found to be equal to the average slope of area A, thus not changing the conclusions.

b. Avalanche time band shown in Fig. 1B

Based on a number of testimonies by the survivors and people they contacted for rescue, and based on the time of the rescue calls registered on the phone devices (information gathered from media reports), the estimated time of the avalanche ranged between GMT 3.48 and 4.08 pm, as drawn in Fig. 1B in Main Text.

REFERENCES

I. Chiambretti, B. Chiaia, B. Frigo, S. Marello, M. Maggioni, R. Fantucci, M. Bernabei, The 18th January 2017 Rigopiano avalanche disaster in Italy – Analysis of the applied forensic field investigation techniques. In ISSW 2018 International Snow Science Workshop, 7–12 October 2018, Innsbruck, Austria, 1208–1212 (2018).
